# Morbidity and Mortality of Heterotopic Partial Heart Transplantation in Rodent Models

**DOI:** 10.3390/jcdd10060234

**Published:** 2023-05-26

**Authors:** Savannah Skidmore, Morgan A. Hill, Katherine Bishara, Haley Konsek, Jennie H. Kwon, Kelvin G. M. Brockbank, Taufiek Konrad Rajab

**Affiliations:** 1 College of Medicine, Medical University of South Carolina, Charleston, SC 29425, USA; 2 Department of Cardiothoracic Surgery, Medical University of South Carolina, Charleston, SC 29425, USA; 3 Tissue Testing Technologies LLC, North Charleston, SC 29425, USA; 4 Department of Bioengineering, Clemson University, Charleston, SC 29425, USA; 5 Department of Pediatric Cardiothoracic Surgery, Medical University of South Carolina, Charleston, SC 29425, USA

**Keywords:** pediatric cardiac surgery, partial heart transplantation, animal model, congenital cardiac surgery, valve dysfunction, rodent

## Abstract

Unrepairable congenital heart valve disease is an unsolved problem in pediatric cardiac surgery because there are no growing heart valve implants. Partial heart transplantation is a new type of transplant that aims to solve this problem. In order to study the unique transplant biology of partial heart transplantation, animal models are necessary. This study aimed to assess the morbidity and mortality of heterotopic partial heart transplantation in rodent models. This study assessed two models. The first model involved transplanting heart valves from donor animals into the abdominal aortic position in the recipient animals. The second model involved transplanting heart valve leaflets into the renal subcapsular position of the recipient animals. A total of 33 animals underwent heterotopic partial heart transplantation in the abdominal aortic position. The results of this model found a 60.61% (*n* = 20/33) intraoperative mortality rate and a 39.39% (*n* = 13/33) perioperative mortality rate. Intraoperative mortality was due to vascular complications from the procedure, and perioperative mortality was due to graft thrombosis. A total of 33 animals underwent heterotopic partial heart transplantation in the renal subcapsular position. The results of this model found a 3.03% (*n* = 1/33) intraoperative mortality rate, and the remaining 96.97% survived (*n* = 32/33). We conclude that the renal subcapsular model has a lower mortality rate and is technically more accessible than the abdominal aortic model. While the heterotopic transplantation of valves into the abdominal aortic position had significant morbidity and mortality in the rodent model, the renal subcapsular model provided evidence for successful heterotopic transplantation.

## 1. Introduction

Congenital heart disease (CHD) is a serious problem, as it remains the most prevalent congenital disorder [1]. The prevalence of CHD ranges from 8.2 to 9.1 per 1000 live births [2]. Valvular dysfunction represents a significant portion of these babies born with CHD [3]. The optimal strategy to address valvular dysfunction is to surgically repair the valves, but sometimes that is not an option due to dysplastic leaflets, bicuspid valves, or other unrepairable abnormalities. The status quo for heart valve replacements in babies is homograft valve replacements, bioprosthetic valve replacements, Ross pulmonary auto-transplant procedures or orthotopic heart transplantation [4,5]. All these options have their disadvantages. Homografts are cadaveric tissue that has lost the ability to self-repair and grow [4]. Neither homograft valves nor bioprosthetic valves grow with the child, leading to subsequent morbid reoperations to replace the valve as the child grows. These valves are also susceptible to having structural degeneration over time, which requires the child to undergo more operations and have an increased risk of morbidity and mortality [6]. Infants who have to undergo any of these procedures are consistently going to outgrow their replacement valve, or the valve will degenerate and cause complications. Studies show that infants and young children who require valve replacement are subjected to receiving sometimes six or more heart procedures. This is because every one of these procedures increases the risk of morbidity and mortality for these children. While the Ross procedure allows for the growth of the autograft, the procedure has an elevated risk of morbidity and mortality in newborns [7,8]. 

In regard to orthotopic heart transplantation, there are limited donors and recipient pools due to extensive eligibility criteria. Approximately 20–25% of infants on the waiting list die prior to this life-saving procedure [9,10]. Therefore, the major push to address this issue has been dedicated to trying to grow heart valves using tissue engineering [11]. Unfortunately, with the current technology, these efforts have not shown any promising results for clinical translation in the near future [12]. All of these issues demonstrate that there is a need for alternative treatment options for children with congenital valve dysfunction.

Partial heart transplantation presents a new type of transplant procedure that has the potential to improve patient outcomes and decrease the need for repeated surgeries across the patient’s lifespan [13]. Partial heart transplantation involves transplanting only the valves of a donor heart into the recipient [14]. The partial heart transplant differs from conventional homografts in that the cells remain viable. Conventional homografts are harvested from cadavers, and this process incurs a long ischemic time that kills the cells in the tissue. In the proposed partial heart transplant procedure, the ischemic time is reduced to ensure a living graft. A living graft has the ability to grow and repair, processes that are essential for the long-term success of the procedure [13]. In a typical orthotopic heart transplant, failure of the transplant almost always occurs due to ventricular dysfunction, while the valves remain functional. In the partial heart transplant, it is irrelevant whether or not the ventricles will function, because only the valves are transplanted. Since evidence from orthotopic heart transplants suggests that the transplanted valves do not fail, partial heart transplantation has an increased likelihood of success in this population [15]. Partial heart transplantation also increases the number of hearts available for donation. Therefore, transplantation would become an option for more pediatric patients with end stage valve dysfunction. There is also a possibility that the valve-specific transplant will be less prone to rejection than a full heart transplant, but this assertation requires more research and assessment of outcomes [16].

A rodent model is necessary for the study of the transplant immunobiology of partial heart transplants. A rodent model can be applicable to the study of human heart transplantation procedures due to similar anatomy and physiology. Rodent models are also more accessible and cheaper than large animal models. In this study, we sought to evaluate rodent models for heterotopic aortic valve transplantation. Gaining knowledge and experience from animal models is essential for the development and optimization of this innovative treatment strategy. 

## 2. Materials and Methods

### 2.1. Animals

This study was approved by the Committee of Animal Research following the National Institutes of Health Guide for Care and Use of Laboratory Animals (MUSC IACUC Protocol ID 2020-01093). All personnel working with the animals had the required course training and certifications. The heterotopic heart valve transplant was tested in young rat pups, with the goal to assess for valve growth over time. Four strains of rodent (Rattus) species were used: Wistar, Sprague Dawley, Lewis, and Norway. Donor and recipient animals were not paired based on species. The immunobiology of valve transplantation suggests that MHC matching may not be necessary for the success of the valve transplant. This experiment utilized transplants between rats of the same species as well as transplants between different species for donors vs. recipients to test this theory. 

Pediatric rats were used in this study to assess the likelihood of success for clinical translation into the congenital human population in the future. The rats used were postpubescent (35 days or greater). While they are not infants, this age group should still be demonstrating valve growth. The growth of the valves is what needs to be studied in order to assess the procedure’s potential success in its intended purpose. 

Thirty-three partial heart transplants using the abdominal aortic model were completed, and thirty-three partial heart transplants using the renal subcapsular model were completed. There were 47 total donor animals. Thirty-three donor animals were used for the abdominal aortic model, donating their entire valve. Fourteen donors were used for the renal subcapsular model, and they each donated 3 leaflets that were separated and used in multiple recipients. Some of the leaflets were damaged in the separation process, which necessitated a few more donors than expected for the 33 transplants. 

### 2.2. Experimental Design

**Figure 1:**
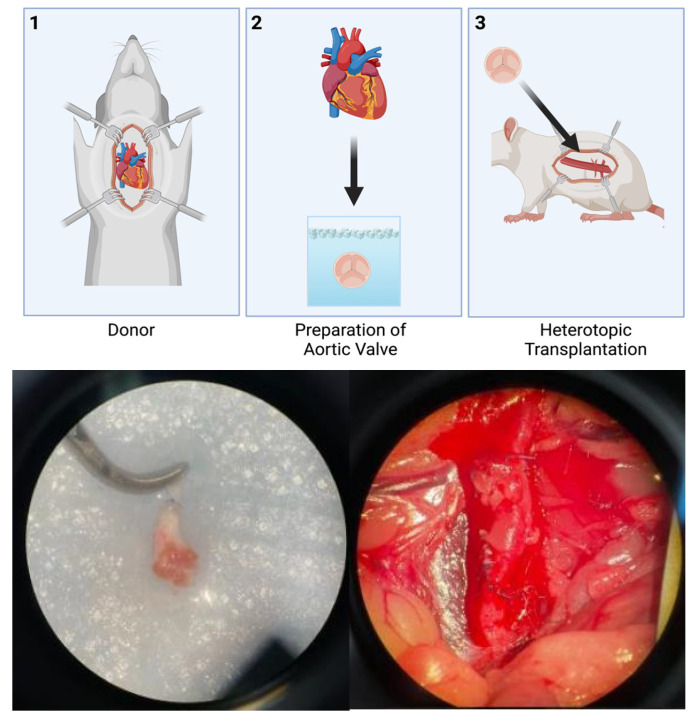
Abdominal Aortic Model for Heterotopic Partial Heart Transplantation, Created with BioRender.com. (**1**) Donor valve is harvested (**2**) the aortic valve is prepared for transplantation (**3**) the valve is transplanted into the abdominal aortic position in the recipient rat. Intraoperative photographs show the donor aortic valve before and after heterotopic transplantation.

**Figure 2:**
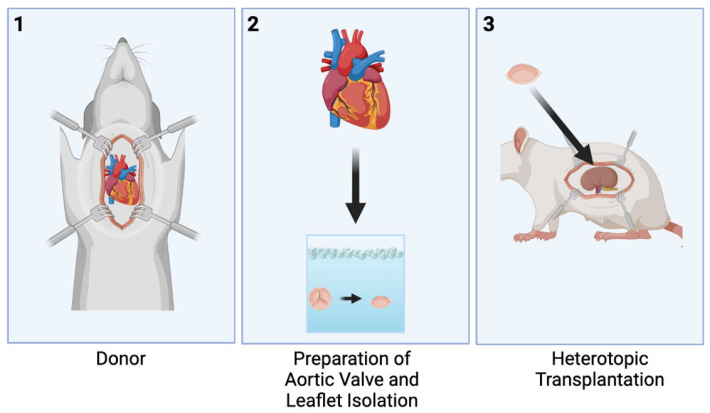
Renal Subcapsular Model for Heterotopic Partial Heart Transplantation, Created with BioRender.com. (**1**) Donor valve is harvested (**2**) the aortic valve is divided into leaflets and prepped for transplantation (**3**) the valve leaflet is transplanted into the renal subcapsular position.

The donor animals underwent valve extraction. The valves were stored in ice-cold University of Wisconsin cardioplegic solution until the time came for transplantation into the recipient animals. Two models were studied. The first model involved transplanting an entire aortic valve from a donor into the abdominal aortic position of the recipient (Figure 1). The second model involved transplanting only one leaflet of an aortic valve from a donor into the renal subcapsular position of the recipient (Figure 2). Intraoperative and perioperative mortality outcomes for each model were calculated. 

### 2.3. Donor Surgical Procedure

First, the donor animal was anesthetized in a dedicated induction chamber. The intraoperative anesthesia used was isoflurane titrated to effect, and it was maintained using a nose cone. The depth of anesthesia was clinically monitored during the procedure by assessing the toe pinch withdrawal reflex, respiratory rate, and observation of movements. A warming mat was used to thermally support the animal intraoperatively. A laparotomy was performed, exposing the inferior vena cava and the abdominal aorta. Next, the animal was heparinized via the vena cava. Intravenous heparin was administered intraoperatively at a dosage of 30 units per kilogram. At this point, the animal was euthanized by exsanguination via aortic transection. The remainder of the procedure was performed on the deceased rat. The incision from the laparotomy was then expanded into a sternotomy. Cold cardioplegic solution was then infused into the ascending aorta, and then the ascending aorta was cross-clamped. The right heart was vented by dividing the inferior vena cava, and the left heart was vented by incising the left atrial appendage. In order to extract the heart, the aorta, pulmonary artery, superior vena cava, inferior vena cava, and pulmonary veins were all divided. The aortic valve and root were then dissected and extracted from the heart and were stored in ice-cold University of Wisconsin cardioplegic solution until time of transplantation.

### 2.4. Recipient Surgical Procedure—Abdominal Aortic Position

First, pre-emptive analgesia was administered via subcutaneous buprenorphine at a dose of 0.01 to 0.05 mg/kg. Next, the recipient animal was anesthetized. The anesthesia used was isoflurane titrated to effect, and it was maintained using a nose cone. The animal was continuously observed for clinical evidence of the surgical plane of anesthesia by assessing the toe pinch withdrawal reflex, respiratory rate, and any movements. A warming mat was used to thermally support the animal throughout the procedure. Then, a laparotomy was performed, and the animal was subsequently heparinized at a dosage of 30 units per kilogram, which was the same as the donor procedure. The abdominal aorta was circumferentially mobilized from below the renal arteries to the bifurcation of the iliac arteries. This segment was isolated by applying vascular clamps. At this point, the aorta was incised, and the donor heart valve was anastomosed to the arteriotomies. The aorta was de-aired by releasing the distal clamp and then the proximal clamp before the last knot was tied down. Lastly, the laparotomy was closed using separate running Vicryl^®^ sutures for the rectus muscle and the deep dermal layer. The epidermis was closed with a running suture reinforced with interrupted Monocryl^®^ sutures.

### 2.5. Recipient Surgical Procedure—Renal Subcapsular Position

For this model, only one leaflet of the aortic valve was required. The donor aortic valve was dissected into the three leaflets, and only one was implanted per recipient animal. The recipient animal was given pre-emptive analgesia in the same manner as the previous model. Buprenorphine was given subcutaneously at a dose of 0.01 to 0.05 mg/kg. Subsequently, the animal was anesthetized using isoflurane titrated to effect, and the anesthesia was maintained using a nose cone. A warming blanket was used for thermal support. The kidney of the recipient animal was exposed using a flank incision. Next, a small incision was made in the capsule of the kidney using Vannas spring scissors. Then, a shallow subcapsular pocket was made using a blunt probe, and the valve leaflet was implanted. The capsule was not routinely closed. Lastly, the flank incision was closed in two layers. 

### 2.6. Postoperative Assessment of Recipient

The postoperative animals were given 0.01–0.05 mg/kg of subcutaneous buprenorphine for pain. The animals were placed in a physiologically prone position. Additional buprenorphine was administered every 6–12 h on the day of surgery and postoperative day 1 and then as needed. The animals were monitored by observing their respiratory rate, whisker movement, and movement of the limbs. Monitoring occurred every 15 min until they emerged from anesthesia, and then after emerging, they were monitored every 6–12 h on the day of surgery. Thermal support was given using a warming mat. Any animals that survived past that point were monitored every 6–24 h as needed. Survival surgery was categorized as one in which the rat regained consciousness. Terminal surgery was categorized as the animal being sacrificed while it was still under anesthesia, never regaining consciousness.

## 3. Statistical Analysis

The abdominal aortic model and the renal subcapsular model were compared using the age of the animal at transplant (for both the donor and recipient), weight of the animal at transplant (for both the donor and recipient), and the ischemic times for the grafts. Continuous variables are presented with standard deviations (SD). Statistical analysis was performed using GraphPad Prism (version 9.3.1 for Mac OS, GraphPad Software). *t*-tests were used to assess if there was a statistically significant difference between the two models. The significance level was set at a *p*-value of 0.05. The variables were compared graphically using error bars, which represent one standard deviation from the mean. 

## 4. Results

### 4.1. Animal Demographics

Abdominal Aortic Position: Pediatric rats, instead of adults, were used to simulate a growing patient. The average age of the donor rats was 69.92 days (±33.31 SD). The average age of the recipient rats was 89.78 days (±32.62 SD). The average donor weight was 292 g (±135 SD). The average recipient weight was 407 g (±121 SD). 

Renal Subcapsular Position: The age difference between donor and recipient was significant. The average age of the donor rats was 57.71 days (±7.21 SD). The average age of the recipient rats was 62.80 days (±9.12 SD). The average weight of the donor animals was 257 g (±57 SD). The average weight of the recipient animals was 238 g (±49 SD). 

**Figure 3:**
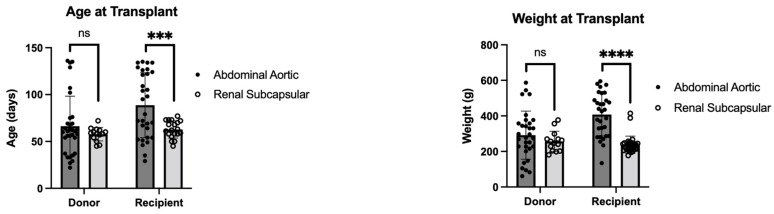
The average age (days) and average weight (grams) of both donor and recipient rodents are compared between the renal subcapsular model and the abdominal aortic model for heterotopic partial heart transplantation. ns = nonsignificant; ***/**** = statistically significant (*p* < 0.05).

The comparison of demographics between the models is depicted in Figure 3. The difference in donor age was found to not be statistically significant (*p* = 0.16). The comparison of recipient age found a statistically significant difference between models, with the abdominal aortic model having an older age at time of transplant (*p* < 0.01). The difference in weight at transplant was nonsignificant between donor animals (*p* = 0.23). The difference in weight at transplant between recipient animals was statistically significant, with the abdominal aortic model having an increased weight at time of transplant (*p* < 0.01).

### 4.2. Graft Ischemic Times

Abdominal Aortic Position: The ischemic time for the grafts of this model was defined as the duration of time from the when the graft was extracted and prepared to the time the clamp was removed from the aorta of the recipient after the completion of the transplant. The average time the clamps were on the aorta was 46 min (±16.30 SD). The average overall graft ischemic time was 2.33 h (±1.35 SD).

Renal Subcapsular Position: The ischemic time for the grafts of this model was defined as the duration of time from when the graft was extracted and prepared to the time of graft implantation in the recipient renal subcapsular space where it regained perfusion. The average ischemic time was 1.09 h (±0.58 SD). 

**Figure 4:**
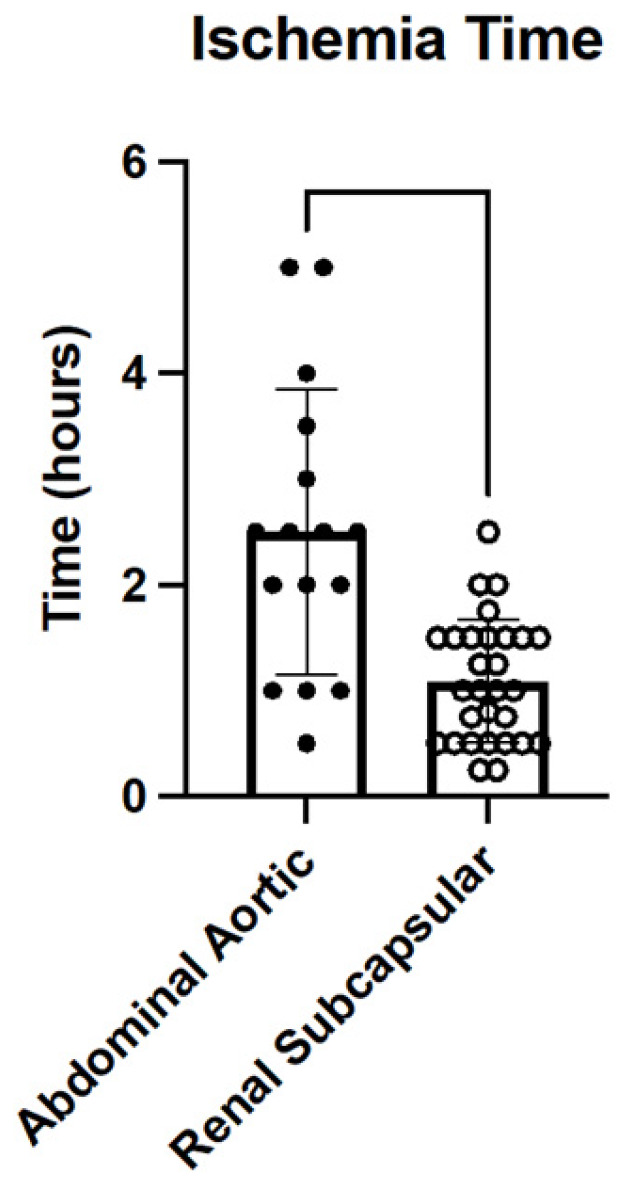
The average ischemic time compared between the renal subcapsular model and the abdominal aortic model for heterotopic partial heart transplantation.

The comparison of graft ischemic times is shown in Figure 4. The difference in ischemic times was found to be statistically significant, with the abdominal aortic model having an increased ischemic time (*p* < 0.01). The ischemic time was unable to be calculated for all transplants due to limits in documentation. Consideration should be taken to address this in future experiments. 

### 4.3. Mortality Results

Abdominal Aortic Position: Thirty-three rats were transplanted using the abdominal aortic model. Twenty of the animals died intraoperatively (intraoperative mortality = 60.61%; *n* = 20/33). Out of the 20 animals that died intraoperatively, 75.00% (*n* = 15/20) of them died due to vascular injury. This included anastomotic bleeding, damage to the inferior vena cava, and clamp injury. The anastomoses in this procedure were technically extremely difficult in such a small animal. There was substantial difficulty in achieving hemostasis. Excessive bleeding occurred in most of the procedures. The five rats that did not suffer vascular injury subsequently developed graft thrombosis, determined by a lack of waveform on the pulse oximetry of the lower extremities. This represented the 25% remainder of intraoperative deaths, as they were subsequently sacrificed (*n* = 5/20). 

Thirteen of the animals survived (woke from anesthesia) and died perioperatively (perioperative mortality = 39.39%; *n* = 13/33). The longest time of survival was noted to be 48 h. Out of the animals that survived, all 13 suffered graft thrombosis. The average time of thrombosis occurred at around 60 min postoperatively. This result was determined by the surgeons noting that the animals lost the ability to move one of their hind legs. Their paralysis was indicative of a lack of blood flow to their extremities due to their graft thrombosis. This assessment was confirmed via autopsy. All the animals that survived and had aforementioned surgical complications were sacrificed under isoflurane by aortic transection.

Renal Subcapsular Position: Thirty-three rats were transplanted using the renal subcapsular model. Only one of the animals died intraoperatively (intraoperative mortality = 3.03%; *n* = 1/33). The animal was noted to have died of respiratory depression from anesthesia before the graft was implanted. The remaining 32 animals survived the procedure (survival rate = 96.97%; *n* = 32/33). All of the surviving animals were noted to have no distress at the 48 h mark. They were found to be awake, eating, drinking, and moving around normally. After their noted successful survival of the operation, the animals were sacrificed via aortic transection under isoflurane. 

## 5. Discussion

In this section, we describe our experience with heterotopically transplanting an aortic heart valve into the abdominal aortic position and aortic valve leaflets into the renal subcapsular space in a rodent model. The abdominal aortic model was unsuccessful and lead to the death of all transplanted animals. This is in contrast to the renal subcapsular model that allowed for a very high survival rate with minimal complications. Therefore, the renal subcapsular model would be more useful for the study and assessment of the immunobiology of heterotopic partial heart transplants. Due to the nature of the procedures, the surgery for the abdominal aortic model requires surgeons to have a significant amount of training, whereas the renal subcapsular model requires very little training for the surgeons. The renal subcapsular space is more readily accessible, with more hemodynamic stability. This is evidenced by the lack of vascular complications in the renal subcapsular model compared to the abdominal aortic position. 

There are two major limitations to the renal subcapsular model. Due to the fact that the valve is dissected and only one leaflet is transplanted, the valve is nonfunctional. While it retains its cellular viability, the valve would never function in the way that it is intended to in this location. In addition, because the valve is not whole and not in physiologic location, valve annulus growth over time cannot be measured. It is essential for the development of partial heart transplantation and its implementation in congenital heart patients that researchers be able to prove growth over time. The major advantage of the renal subcapsular model is that it was successful in demonstrating a low mortality rate and tissue viability. We found the procedure itself to be more technically accessible and to have a much higher rate of intraoperative survival, as compared to the abdominal aortic position. In addition, this procedure allowed us to minimize animal use, as the donor can donate up to three leaflets from their aortic valve, and the subrenal capsule model only requires one leaflet to be implanted. Thus, the model allows for three recipients for every one donor [17].

The abdominal aortic model was unsuccessful for several reasons. First, as mentioned earlier, the abdominal aortic procedure is extremely technically difficult, as evidenced by the 60% intraoperative mortality rate. The location of implantation, the abdominal aorta, is an area of high blood flow that leads to serious complications if hemorrhage occurs. The next complication that needs to be addressed is the graft thrombosis, which occurred in 54.55% (*n* = 18/33) of the operations. We believe that this occurred due to the donor valve being placed in series with the native valve. The pressure gradient required to open and close the native (proximal) valve is inherently absent for the distal valve. This causes the absence of leaflet motion, predisposing to clot formation. The surgical procedure as it stands causes significant endothelial damage to the vessel walls, which can contribute both to the issues obtaining hemostasis, as well as clot formation after hemostasis has occurred [18]. Graft thrombosis could potentially be addressed in several ways. First, anticoagulation therapy could be used to decrease the degree and prevalence of thrombosis observed in this study. This solution is not an ideal one due to the propensity for bleeding and hemodynamic instability. Another, and possibly safer, solution to the problem would be to render the native valve incompetent. This would alleviate the pressure gradient issue. However, this is technically difficult. The last option would be to use a larger animal model with an orthotopic transplant instead of a heterotopic one [19]. Research suggests that piglets would be a good model due to their anatomical and physiological similarities to humans [20]. The piglets planned for use would have larger hearts and vessels than rats, which would subsequently lead to a more accurate representation of the surgical challenges and physiological responses to the procedure in humans. 

Many of the limitations of the abdominal aortic model that led to high mortality and morbidity rates were due to the surgical difficulty of the procedure itself, as well as the model and study design not being optimal for long-term assessment. The ischemic time of the abdominal aortic position model is longer because it is technically far more demanding. However, the ischemic time is still within the clinically acceptable limits for partial heart transplants. One major difference between this study and previous ones is that instead of working with adult rats, this model requires that the operations occur in pediatric rats. Pediatric rats were used to allow the evaluation of partial heart transplant valve growth over time. We selected recipient rats that were older and larger than their counterparts to assist in the difficulty of the procedure by size matching the donor aortic root with a smaller recipient abdominal aorta. This difference is evident in the statistical difference depicted in Figure 3. The increase in age and weight was not enough to compensate for the surgical risk. This study incurred a higher mortality rate than previous studies using the abdominal aortic model in adult rats because of the size difference in pediatric rats [21,22]. In addition, there was a learning curve for the surgeons performing the procedures, which led to the rats in the earliest experiments being the most likely to die intraoperatively from aortic or inferior vena cava injury. Later on, death was more likely perioperatively from graft thrombosis. Both surgeons had significant training on the procedure; however, the operation on such a small model had an adjustment period. Another limitation is that the heterotopic method of transplantation is required in this model because there are no suitably small heart–lung machines available for an animal of this size. The change to an orthotopic procedure, which would inevitably require a larger animal model, would provide greater insight into the true valve function. 

With regards to clinical translation, the understanding of the immune response to the partial heart transplant is essential in planning for the immunosuppressive regimens required for patients. The current literature suggests that heart valves may have some degree of immune privilege that is comparable to other areas, such as the central nervous system, eyes, testes, placenta, and cartilage [16]. The evidence for this lies in the fact that when an orthotopic heart transplant is rejected in patients, evaluation of the valves shows intact function with minimal regurgitation or stenosis [23,24]. This suggests that the valves are in some way immunologically distinct from the cardiac muscle tissue and potentially more immune to the body’s transplantation rejection process. In order to study the transplant immunobiology of partial heart transplants, other studies found greater success with changing the location of implantation to the subrenal capsule. A better understanding and assessment of the immunobiological properties and the potential for growth after the proposed procedure need to be evaluated in future studies. 

## 6. Conclusions

While the heterotopic transplantation of valves into the abdominal aortic position had significant morbidity and mortality in the rodent model, the renal subcapsular model provided evidence for successful heterotopic transplantation. We still believe that larger animal models will be pivotal for studying the immunobiology and growth potential of this new type of transplant procedure. More research needs to be conducted to adjust the methodology and the model to achieve optimal postoperative survival and assessment. The goal moving forward will be to demonstrate the success of the procedure in a larger, more comparable model to humans. In future studies, the growth of the valves over time should be measured, all with the goal of improving outcomes and quality of life for patients with congenital valve dysfunction.

## Data Availability

The data are contained within the article.
